# Editorial: Rising stars: Bone research 2021

**DOI:** 10.3389/fendo.2022.1114664

**Published:** 2022-12-15

**Authors:** Melanie Haffner-Luntzer, Ahmed Al Saedi

**Affiliations:** ^1^Institute of Orthopedic Research and Biomechanics, Ulm University, Ulm, Germany; ^2^Division of Endocrinology, Boston Children’s Hospital, Harvard Medical School, Boston, MA, United States

**Keywords:** bone research, bone regeneration, osteosarcoma, fracture, osteoporosis, inflammation

## Introduction

We are delighted to present the Rising Stars: Bone Research 2021 article collection. This collection showcases the high-quality work of internationally recognized researchers in the early stages of their independent careers. All Rising Star researchers were individually nominated by the Frontiers in Endocrinology editors in recognition of their potential to influence the future directions in their respective fields. The work presented here highlights the diversity of research performed across the entire breadth of bone research.

Healthy bones are indispensable for the body’s movement, which is a basic requirement for mobility and participation in everyday life. Mobility is something most people take for granted until it is lost due to bone fractures caused by aging, osteoporosis or cancer. Maintaining healthy bones throughout life, even as people age, is therefore a major challenge. Patients with fractures experience pain, reduced mobility and a significantly reduced quality of life. In addition to the bone’s conventional functions such as support, movement and protection, the skeleton also contributes to whole body homeostasis and maintenance of multiple important non-bone organs/systems (extra skeletal functions). This motivates bone researchers from around the world to investigate the interplay between endocrine and inflammatory systems and bone as well as fracture healing, analyze the influence of specific medication like glucocorticoids on the skeleton, develop novel diagnostic and therapeutic algorithms and determine genetic and non-genetic reasons from bone diseases. The research presented in these research topics bridges the gap between basic research using *in vitro* systems and mouse models to more translational research and up to clinical research.

## Isolation and *in vitro* characterization of murine long bone skeletal progenitors

Skeletal stem and progenitor cells (SSPCs) has been shown in recent years to be important for bone development, maintaining bone health and successful bone regeneration. However, *in vitro* characterization of the different sub-populations is still challenging. Therefore, Loopmans et al. developed a comprehensive flow cytometry-based procedure to isolate, culture and characterize SSPCs from metaphseal bone and endosteum of young-adult mice. These cells possess self-renewing capacity and ability to differentiate into osteoblasts and adipocytes, while differentiation into chondrocytes is limited. This protocol could be used in further studies to study SSPC biology *in vitro* in more detail.

## Myeloid cell-derived catecholamines influence bone turnover and regeneration

The fact that neuroendocrine circuits and bone tissue interacts with each other has gained more and more attention during the last decade. Kuhn et al. showed in their latest manuscript, that besides systemic adrenal and sympathetic catecholamine production, also myeloid immune cells are capable of synthesizing catecholamines, therefore influencing bone cells. They demonstrated that mice lacking catecholamine producing specifically in myeloid cells display an age-dependent decreased bone mass phenotype and delayed fracture healing, based on disturbed osteoblast activity. This indicates a crucial role of myeloid cell-derived catecholamines in immune cell-bone cell crosstalk and during bone regeneration and opens the possibility of novel therapeutics strategies to counteract delayed bone healing.

## Hypomineralization in auditory ossicles of vitamin D receptor deficient mice

Intact mineralization of the auditory ossicles is essential for sound transmission in the middle ear, while ossicular hypomineralization is associated with conductive hearing loss. Delsmann et al. found hypomineralization in auditory ossicles in mice lacking the vitamin D receptor. Furthermore, they determined that this phenotype could be partially rescued by a calcium-rich diet. These results open new treatment strategies for conductive hearing loss in patients with genetic mutations in the vitamin D gene.

## Complement receptor C5aR1 on osteoblasts regulates osteoclastogenesis in postmenopausal osteoporosis

Evidence has accumulated that the complement system, an integral part of innate immunity, may be involved in the regulation of bone homeostasis as well as inflammatory bone loss. Bülow et al. investigated the role of the anaphylatoxin receptor C5aR1 on osteoblasts and osteoclasts during the development of postmenopausal osteoporosis. They found that C5aR1 on osteoblasts is crucial for the induction of bone resorption under osteoporotic conditions by stimulating RANKL release, whereas C5aR1 on osteoclasts did not regulate OVX-induced bone loss. These results implicate that C5aR1 on osteoblasts could be a potential target for treating postmenopausal osteoporosis.

## Level-specific volumetric BMD threshold values for the prediction of incident vertebral fractures

Osteoporotic vertebral fractures are one of the most common and clinically relevant fracture sites, accounting for 16% of reported fragility fractures and 53 – 65% of fracture-related deaths in the EU. A considerable number of patients does not receive existing treatment strategies, partly because of insufficient diagnostic techniques. Therefore, Dieckmeyer et al. evaluated the diagnostic accuracy of volumetric bone mineral density threshold values at different spinal levels for the prediction of incident vertebral fractures. They propose level-specific vBMD threshold at the thoracolumbar spine to identify individuals at high fracture risk.

## Prevention and treatment of glucocorticoid-induced osteoporosis in adults

Glucocorticoids are effective immunomodulatory drugs. However, their long-term use is also associated with several side effects including an increased risk of osteoporosis and fractures. Laurent et al. described in their manuscript the latest recommendations from the Belgian bone club for prevention and treatment of glucocorticoid-induced osteoporosis in adults.

## The genetic overlap between osteoporosis and craniosynostosis

Genome-wide association studies (GWAS) have pinpointed hundreds of loci associated with bone mineral density (BMD), helping elucidate the underlying molecular mechanisms and genetic architecture of fracture risk. Kague et al. describes in their review article the genetic contribution to osteoporosis and how this overlaps with craniosynostosis. They review current knowledge gained from human GWAS studies and zebrafish models.

## Knowledge atlas and emerging trends on ncRNAs of osteosarcoma: A bibliometric analysis

Research on non-coding RNAs (ncRNAs) of osteosarcoma has been developed rapidly in recent years; a specific bibliometric analysis on this topic has not yet been performed. The bibliometric analysis aims to summarize knowledge atlas, research hotspots, and emerging trends and to provide researchers with new perspectives in further studies. Wang et al. offers in their study a scientific perspective on ncRNAs of osteosarcoma and provides researchers with valuable information to understand the knowledge structure and to identify emerging trends in this field

## Conclusion

Research in the area of bone biology, genetics and regeneration is a constantly evolving area with a focus to design effective diagnostics and therapeutics for the management of osteoporosis and fractures. The eight contributions to this topic highlighted the progress and developments of potentially new treatment options, treatment monitoring, collaboration and improvement of care in cancer and bone diseases.

## Author contributions

All authors listed have made a substantial, direct, and intellectual contribution to the work and approved it for publication.

